# Material strengths of shear-induced platelet aggregation clots and coagulation clots

**DOI:** 10.1038/s41598-024-62165-1

**Published:** 2024-05-20

**Authors:** Dongjune A. Kim, David N. Ku

**Affiliations:** Georgia Institute of Technology, G.W. Woodruff School of Mechanical Engineering, 315 Ferst Drive NW, IBB 2307, Atlanta, GA 30332 USA

**Keywords:** Computational biophysics, Cardiovascular biology, Cardiovascular diseases, Heart failure, Biomedical engineering, Biomaterials

## Abstract

Arterial occlusion by thrombosis is the immediate cause of some strokes, heart attacks, and peripheral artery disease. Most prior studies assume that coagulation creates the thrombus. However, a contradiction arises as whole blood (WB) clots from coagulation are too weak to stop arterial blood pressures (> 150 mmHg). We measure the material mechanical properties of elasticity and ultimate strength for Shear-Induced Platelet Aggregation (SIPA) type clots, that form under stenotic arterial hemodynamics in comparison with coagulation clots. The ultimate strength of SIPA clots averaged 4.6 ± 1.3 kPa, while WB coagulation clots had a strength of 0.63 ± 0.3 kPa (p < 0.05). The elastic modulus of SIPA clots was 3.8 ± 1.5 kPa at 1 Hz and 0.5 mm displacement, or 2.8 times higher than WB coagulation clots (1.3 ± 1.2 kPa, p < 0.0001). This study shows that the SIPA thrombi, formed quickly under high shear hemodynamics, is seven-fold stronger and three-fold stiffer compared to WB coagulation clots. A force balance calculation shows a SIPA clot has the strength to resist arterial pressure with a short length of less than 2 mm, consistent with coronary pathology.

## Introduction

**Figure 1:**
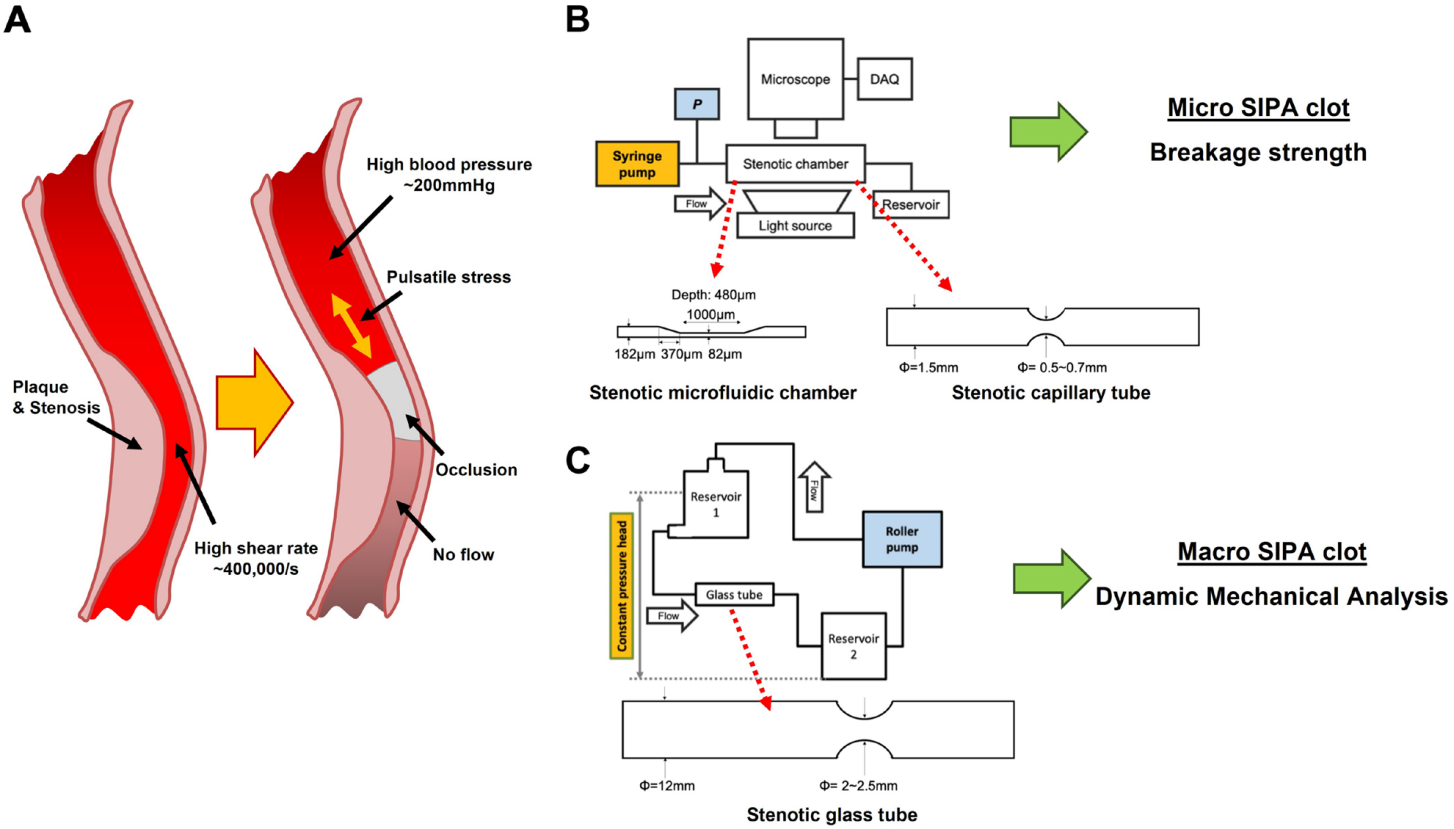
Occlusive arterial thrombosis and in vitro flow loops for studying Shear-Induced Platelet Aggregation (SIPA) clot. (**A**) SIPA clot occluding an artery under high shear rate 1, high blood pressure, and cyclic stress conditions. (**B**) A single-pass flow driven by a constant flow rate with the stenotic capillary tube and microfluidic chamber to measure clot breakage strength. (**C**) A closed loop circuit to generate a SIPA clot in a large stenotic glass tube that has stenosis diameter greater than 2 mm, in order to generate large SIPA thrombi.

Thrombotic occlusions of arteries are leading causes of death from stroke and heart attacks in the United States and worldwide. The pathology of cerebral vascular accidents can often be attributed to the occlusion of blood flow by a clot (thrombosis) located at a narrowing (stenosis) in the artery. Note that this paper does not address embolic phenomenon which can also cause stroke. The sudden formation of an occlusive thrombus in these arteries blocks blood flow and deprives organs of oxygen, resulting in the patient’s death. Arterial thrombi form under high shear rates up to 400,000 s^-1^^1^ and arterial blood pressure over 175 mmHg^2^ (Fig. 1A). For these hemodynamic conditions, the formation of an arterial thrombus material is daunting. To occlude, the growing thrombi adherent to the collagen at the site of a plaque cap rupture must resist being ripped off by the high velocity of viscous blood, and then further resist the high systolic arterial blood pressure to stop blood flow.

Currently, the available treatments for arterial thrombosis are thrombolytic drugs or a thrombectomy for ischemic stroke, and thrombectomy is becoming the more effective treatment as it provides a higher recanalization rate compared to tissue Plasminogen Activator (tPA) treatment alone^3^. Thrombectomy devices such as stent or coil retrievers are wire-based devices that apply mechanical force to break and retrieve a clot. Thus, measurement of the mechanical strength of the clot may be used to improve the success of mechanical thrombectomy devices. An occlusive thrombus may become deformed, elongated, thinned, fractured, and fragmented during thrombectomy^4^. Yeo et al.^5^ suggested that successful thrombectomy depends on clot composition and properties, such as friction^6^, maturity^7^, and stickiness^8^. Thromboelastography (TEG) has been used to measure clot strength and predict clinical outcome of ischemic stroke^9^ and coronary artery disease patients^10^. However, TEG only indirectly measures coagulation clot strength using the unphysiological small angle oscillations of a rod in a clot to measure resisting force. Many studies have attempted to measure the mechanical strength of a blood clot using various methods, including a tensile tester, compression tester, dynamic mechanical analyzer, rheometer, dynamic ultrasound viscoelastography, nanoindentation, and a viscometer^11^. However, these measurements have focused on coagulation clots and resulted in very distinctive values over 10 orders of magnitude (Suppl Fig. 1). The tested coagulation clots were either collected^12–16^ or generated via in vitro assays^17–25^.

There are different types of blood clots (Table 1). Whole blood (WB) coagulation clots and Shear-Induced Platelet Aggregation (SIPA) clots differ in terms of their formation mechanism and composition. A WB coagulation clot follows the classic Virchow’s triad, which consists of endothelial disruption, stagnant blood flow, and hypercoagulability^26^. The main structural component of a WB coagulation clot is fibrin, which is the final product of the coagulation cascade that entraps large amounts of RBCs. A WB coagulation clot is often referred to as a “red clot” because it appears to be red in color due to the presence of a large number of RBCs.

**Table 1:** Types of blood clots.

	WB* coagulation clot	PRP** coagulation clot	SIPA*** clot
Mechanism	Coagulation	Coagulation	Shear-induced platelet aggregation
Flow condition	Static	Static	High shear rate
Main components	Red blood cell, Fibrin, Sparce Platelets	Fibrin, Platelet	VWF Platelet
Color	Red	Pale	White

*WB whole blood.

**PRP platelet-rich plasma.

***SIPA shear-induced platelet aggregation.

In contrast, platelet-rich SIPA clots form under high shear conditions in situ at a stenosis^27^. Casa et al.^28^ hypothesized that an alternative triad is needed for SIPA clot formation: (i) a collagen surface or other substrate for initial von Willebrand Factor (VWF) absorption, (ii) a pathologically high shear rate for VWF unfolding and elongation, and (iii) platelets and VWF in sufficient concentrations. Thus, a SIPA clot is mostly composed of platelets and VWFs: Ku and Flannery’s study^29^ estimated 80% of SIPA clot consisted of platelets by histology, and Kim and Ku’s study quantified a similar amount by scanning electron microscopy of the platelet-VWF-rich SIPA clot structure^30^. SIPA clots look white and are commonly called “white clots” because of the prevalence of platelets and the lack of RBCs^27^. To induce SIPA, many thousands of GPIb-A1 bonds are required to capture platelets under high shear^31^ and high permeability of the clot helps to resist flow forces^32^. These platelet-VWF bonds may give a SIPA clot a high level of mechanical strength so that it can resist the arterial shear rate and blood pressure. In the present study, we evaluate the hypothesis that a thrombus must have sufficient strength and size to withstand arterial pressures to occlude blood flow. We created SIPA clot using in vitro flow systems, measured the strength of SIPA and WB coagulation clots, and analyzed whether either of these clots were strong enough to block arterial blood flow. In addition, we generated Platelet-Rich Plasma coagulation (PRP) clots that are lacking RBC but formed by coagulation^37^ (Table 1). We used PRP for the Dynamic Mechanical Analysis (DMA) to study whether the lack of RBC impacts mechanical strength. PRP coagulation clots made from platelets bound by fibrin provides a possible intermediate case for comparison with WB coagulation clots that are made from RBC and fibrin, as well as for comparison between platelet rich coagulation clots bound by fibrin and platelet rich SIPA clots bound by VWF.

In this study, we compare the mechanical properties of SIPA clots to both WB coagulation clots or PRP coagulation clots formed under stagnant conditions. The mechanical measurements show that SIPA clots are stronger than WB coagulation or PRP coagulation clots. A force balance analysis shows that only SIPA clots are strong enough to block arterial pressures to cause blood flow occlusion.

## Methods

### In vitro stenotic chambers

Three different stenotic chambers (Suppl Fig. 2) were used to induce high shear conditions and generate SIPA clots of varying shapes and sizes. The three chambers are: (1) microfluidic 98% stenosis by height (microfluidic chamber), (2) small artery 85% stenosis by diameter (stenotic capillary glass tube, ID = 1.5 mm), and (3) large artery 45% stenosis by diameter (stenotic large glass tube, ID = 12 mm) in diameter. Prior to the blood perfusion, the stenotic region of each chamber was coated with type 1 fibrillar collagen and incubated in a container for 24 h to generate an adhesive surface. The 12 mm large tube (Suppl Fig. 2C) was rotated 180 degrees once after the initial hour.

### In vitro flow loops for SIPA clot generation

To generate a SIPA clot, porcine whole blood was perfused through three in vitro flow systems with the three different stenotic chambers. The use of light heparin (3.5 IU/mL) as anticoagulant for SIPA generation was established by Para et al^33^ and Claveria et al.^34^ as superior to citrate in yielding rapid occlusion, since citrate can affect platelet activity. All methods were carried out in accordance with relevant guidelines and regulations. We use blood harvested from farm pigs after sacrifice. Thus, no live vertebrates are studied, and the Institutional Animal Care and Use Committee does not regulate the studies using blood from an abbatoir. Silicone (PDMS, Polydimethylsiloxane) microfluidic and glass capillary tubes were used in single-pass in vitro flow systems with constant flow. For the constant flow experiments (Fig. 1B), the flow rate was controlled using a syringe pump to generate an initial wall shear rate of 3500 s^−1^ in the stenosis^1,33^. The pressure proximal to stenosis was monitored via a pressure transducer using Labview software. SIPA clot formation in the stenosis was recorded throughout the experiment using a high- resolution CCD microscope camera. Meanwhile, a high flow rate (~ 1 L/min) was required to generate high shear in the large tube (Fig. 1C). To accommodate this requirement, a closed in vitro flow loop was developed with two reservoirs for a constant pressure head (30 mmHg) (Fig. 1C). As the clot grew in the tube, the flow rate of the roller pump was manually reduced every 1–2 min and recorded to keep the upstream reservoir from overflowing. When the roller pump flow rate reached a minimum level (0.05 L/min), the pump was turned off and left in the circuit for an additional 30 min to achieve the full occlusion. More details regarding the flow system such as pump control and chamber manufacturing can be found in the paper by Kim and Ku^30^.

### Whole blood and platelet-rich plasma coagulation clot generation

Two types of coagulation clots were formed: (1) Whole blood (WB): A non-stenotic capillary tube was coated with type 1 fibrillar collagen and incubated in a container for 24 h to generate an adhesive surface. Another set of tubes were left uncoated. Porcine blood was treated with 3.2% sodium citrate (10% in volume) during transportation and recalcified with CaCl_2_ to a final concentration of 10 mM just before placing the blood in the capillary tube based on an established technique for creating WB coagulation clots^34–36^. 20 μl of re-calcified porcine whole blood was placed in the capillary tube and left stagnant for at least 30 min to form a stable WB coagulation clot prior to the perfusion studies.

(2) Platelet-rich plasma (PRP): These non-physiologic, platelet-rich clots formed under static conditions provides a strong contrast to SIPA clots, even though the platelet content is similar. For PRP coagulation clot generation, we first made PRP by collecting the supernatant of the centrifuged citrated whole blood via gravity over 2 h period. Using citrated PRP, we generated a PRP coagulation clot under stagnant conditions. The PRP coagulation clot is composed primarily of platelets under static conditions and is connected by fibrin instead of VWF^37^.

### Breakage strength measurement

**Figure 2:**
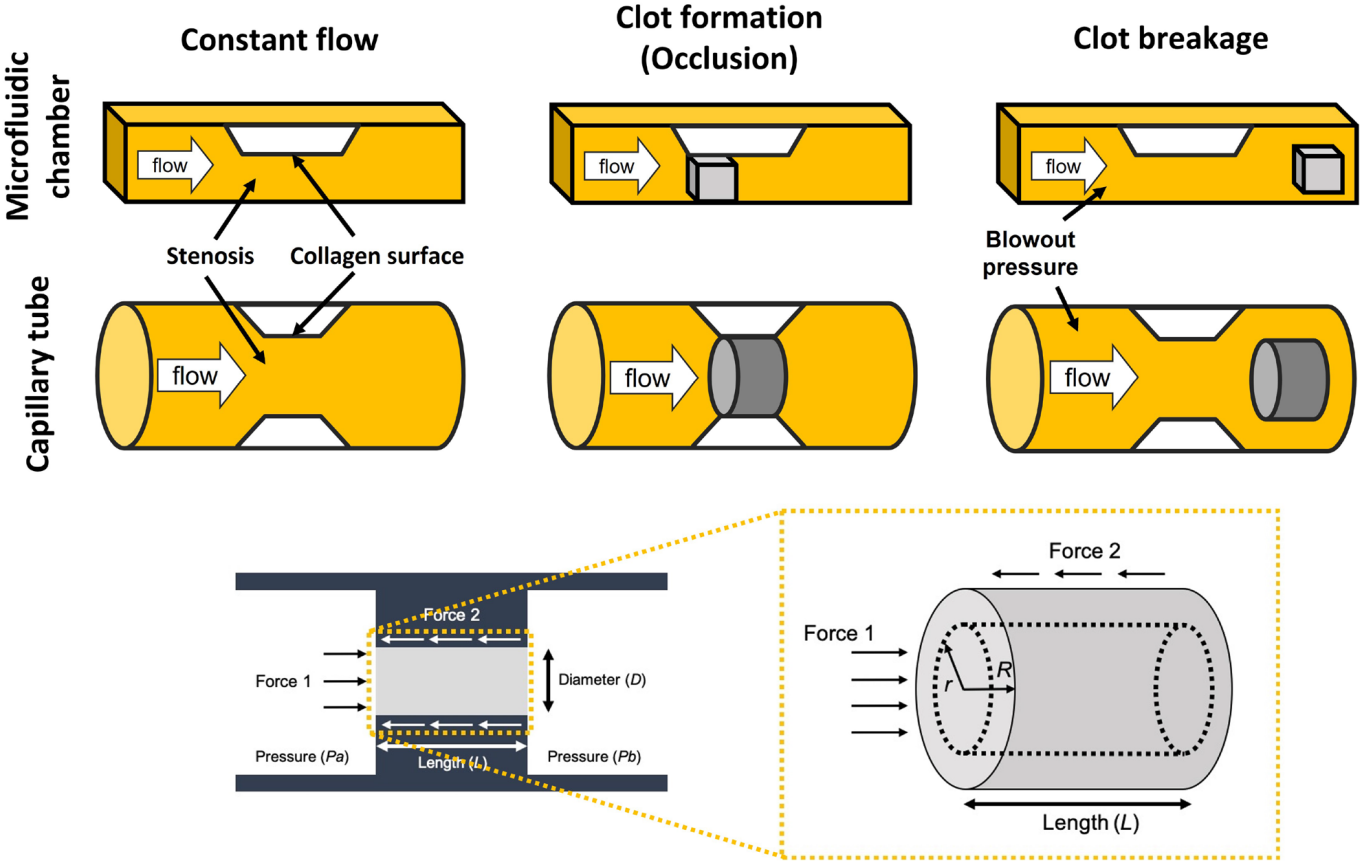
Forces acting on an occlusive clot in a stenotic chamber. (**A**) Two- dimensional and (**B**) three-dimensional views of forces and parameters on an occlusive clot.

With a syringe pump, constant flow was induced into the stenotic chambers to generate a SIPA clot. The pressure upstream increased as the SIPA clot grew at the stenotic region; eventually, the clot broke off abruptly and this pressure (blow-out pressure) was later used to calculate the breakage strength. There are assumptions we made to estimate the clot breakage strength: (1) The clot is occlusive and fully occupies the channel lumen. Thus, the SIPA clot in a microchannel is modeled as hexahedron while a capillary tube is modeled as cylinder shape. (2) The clot is homogenous. (3) No visco-elastic deformation was considered as the breakage occurred in a very short period of time, and the thrombus broke near the chamber wall. (4) After the breakage, the SIPA clot regrew and reoccluded the chamber suggesting that collagen remained on the glass. Therefore, the broken part was not the collagen-glass wall interface (Fig. 2).

Upon these assumptions, the force balance acting on the clot formed in the capillary tube are illustrated in Fig. 2 and calculated as follows:

$${F}_{1}=\pi {r}^{2}\bullet \Delta P$$

$${F}_{2}= \pi r\bullet L\bullet \tau$$

where *r* denotes radial distance, *∆P* is the pressure difference across the clot (*Pa-Pb*), *L* is the clot length, and *τ* is shear stress. The balance between these two forces, *τ*, was calculated using the following equation:

$$\tau =\frac{r\bullet \Delta P}{2L}$$

**Figure 3:**
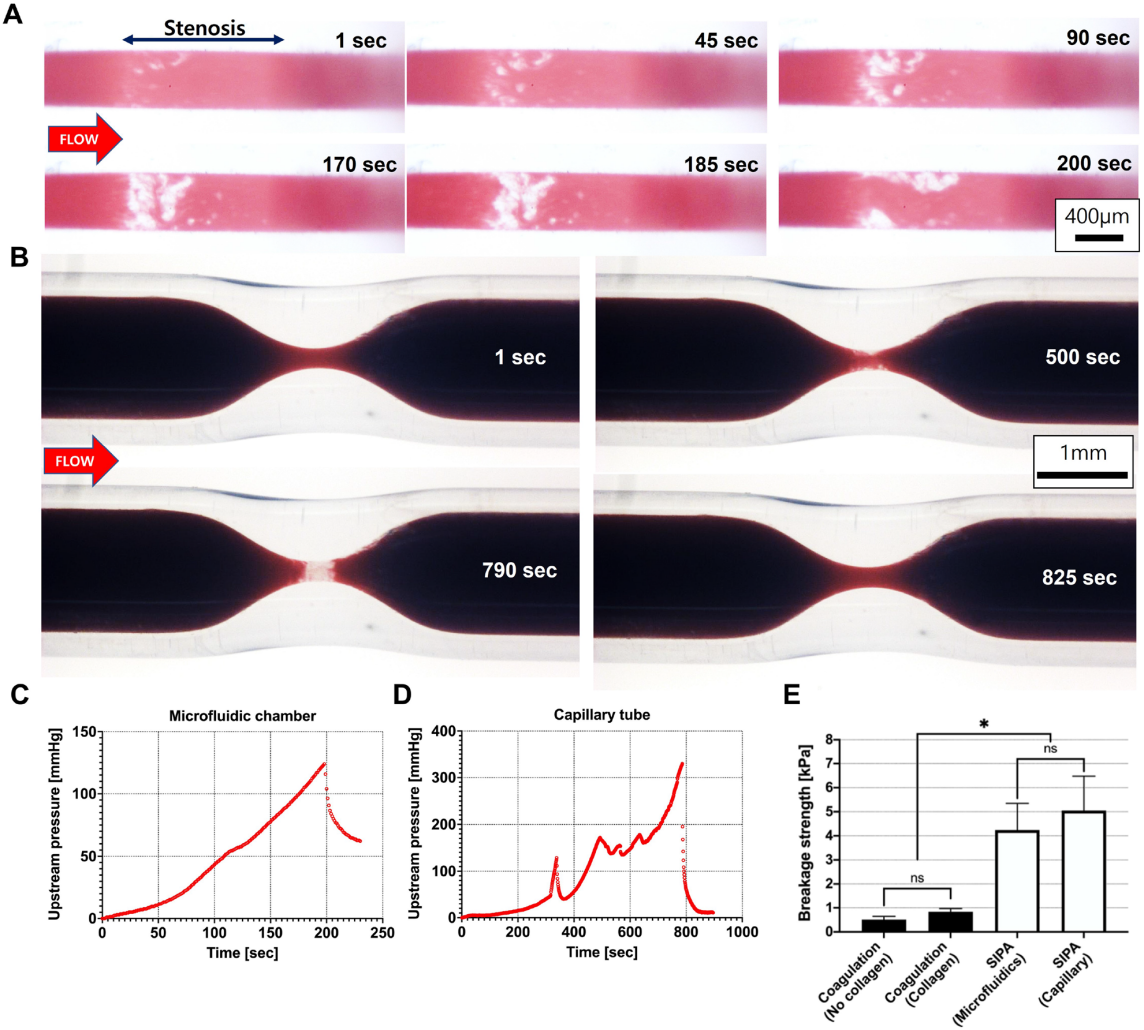
Shear-Induced Platelet Aggregation (SIPA) clots have significantly higher breakage strengths compared to Whole Blood (WB) coagulation clots. (**A**) Shear-Induced Platelet Aggregation (SIPA) clot formed in a stenotic microfluidic chamber over time and eventually broke between 185 ~ 200 s. (**B**) SIPA clot formed in a stenotic capillary tube occluded stenosis (790 s) and embolized downstream (825 s). (**C** and **D**) Upstream pressures for (**A**) and (**B**) increased over time and dropped abruptly when the SIPA clots embolized. (**E**) SIPA clots showed significantly higher breakage strength (*p < 0.05, n = 7) than the WB coagulation clots. The presence (n = 3) or absence (n = 3) of a collagen surface did not significantly impact WB coagulation clot strength. The type of stenotic chamber did not significantly change SIPA clot mechanical breakage strength for a microfluidic chamber (n = 7) or a capillary tube (n = 7).

The maximum stress occurs at the most-outer edge (*r* = *R*) of the clot; this was confirmed in the experiments, as the clots were found to be broken as one whole piece (Fig. 3). The following equation was used to calculate the breakage strength of the clot (τ_MAX_):

$${\tau }_{MAX}=\frac{R\bullet \Delta P}{2L}$$

The same force balance principle is applied to microfluidic chamber and equation can be written as follows:

$${\tau }_{MAX}=\frac{h\bullet w\bullet \Delta P}{2(h+w)L}$$

where *h* is the channel height and *w* is channel width. All geometrical parameters (*R, L, h, w*) were measured from clot image and pressure gradient was measured via pressure data upstream (*P*_*b*_ = 0).

### Dynamic mechanical analysis

We used three types of clots: SIPA, WB coagulation, and PRP coagulation, for the Dynamic mechanical analysis (DMA)^38^ to see if different clot components (Table 1) had differing mechanical properties. DMA^38^ was performed on retrieved blood clots from the large glass tube (Fig. 1C), the WB coagulation clot, and the PRP coagulation blood clot using an ElectroForce 3100 (TA instruments, New Castle, DE) materials testing machine. Three displacements (0.1 mm, 0.2 mm, and 0.5 mm) and three different frequencies (0.1 Hz, 1 Hz, and 10 Hz) were applied to observe the displacement dependency and quantify the frequency response. Displacement conditions were chosen to collect good signals, and frequencies were selected around the physiological heat beat frequency (1 Hz). The compressive modulus was calculated based on the cross-sectional area of each clot and, the magnitude of displacement curve, and the force response curve per the general definitions for DMA^39^.

### Computational fluid dynamics and finite element analysis

Fluid–structure interaction (FSI) technique is used to simulate the pressure distribution in the stenotic glass tube with SIPA clot during blood flow. The solid thrombus deformation and stress was calculated by finite element (FE) analysis. The simulation was performed using Ansys 19.1 (Ansys Inc., PA, USA, https://www.ansys.com/). To simulate arterial blood flow, whole blood flow was assumed to be a Newtonian fluid of 3.5 cP, and the flow was presumed to be laminar, incompressible, steady, continuum, and isothermal^1^. The no-slip boundary condition was applied to the walls, and a 30 mmHg pressure was applied at the inlet with zero pressure at the outlet. Mesh convergence was achieved at 2.6 million hexagonal cells. The clot is modeled as a porous media. The FE analysis was simplified by applying a 2-D axisymmetric condition. A fixed support boundary condition was applied at the interface between the clot and channel wall and the pressure distribution from CFD results was applied at the upstream and downstream clot surfaces. For FE analysis, mesh convergence was achieved at 0.1 million tetragonal cells.

### Data analysis

A one-way ANOVA was used to test for statistical differences between groups with the significance set at p < 0.05 (GraphPad Prism 7, GraphPad Software, San Diego, CA, https://www.graphpad.com/). All data are presented as the mean ± standard deviation unless otherwise noted. Data availability: The original datasets used and/or analyzed during the current study are available from the corresponding author on reasonable request.

## Results

### SIPA clot has greater breakage strength than WB coagulation clot

Whole blood was perfused through stenotic chambers using a syringe pump, driving a constant flow rate. At the stenosis, SIPA clots started to form with white color (Fig. 3A and B) and grew over time to occupy each flow chamber. Upstream pressure increased over time as SIPA clots grew and became occlusive (Fig. 3C and D). For continuous flow pumps, the upstream pressure can continue building to an unphysiologic level of thousands of mmHg. We used this feature to establish a measure of the strength of the occluding clot. At a certain point, the SIPA clot abruptly broke (e.g. between 185 ~ 200 s for microfluidics chamber or 790 ~ 825 s for capillary tube) and either was hanging in the chamber be a thread (Fig. 3A) or completely embolized downstream (Fig. 3B). The breakage of the clot matched a dramatic pressure drop at the upstream (Fig. 3C and D). We then used the peak pressure to calculate SIPA clot breakage strength. The same method was applied to measure WB coagulation clot strength using non-stenotic capillary glass tubes. On average, SIPA clot resisted a pressure gradient of 160 mmHg for microfluidics and 260 mmHg for capillary tubes. In turn, the SIPA clots demonstrated a breakage strength of 4.6 ± 1.3 kPa that was 7 times significantly stronger than the WB coagulation clots with a breakage strength of 0.63 ± 0.3 kPa (Fig. 3E, p < 0.05). Both stenotic chamber revealed similar breakage strengths of the SIPA clots (microfluidic vs. capillary tube). After the breakage, the SIPA clot regrew and reoccluded the chamber suggesting that collagen remained on the glass. Therefore, the broken part was not the collagen-glass wall interface. For WB coagulation clot experiments, the surface with or without collagen type 1 did not significantly impact the breakage strength of these WB coagulation clots.

### A SIPA clot is stiffer than a coagulation clot with (WB) or without RBC (PRP)

**Figure 4:**
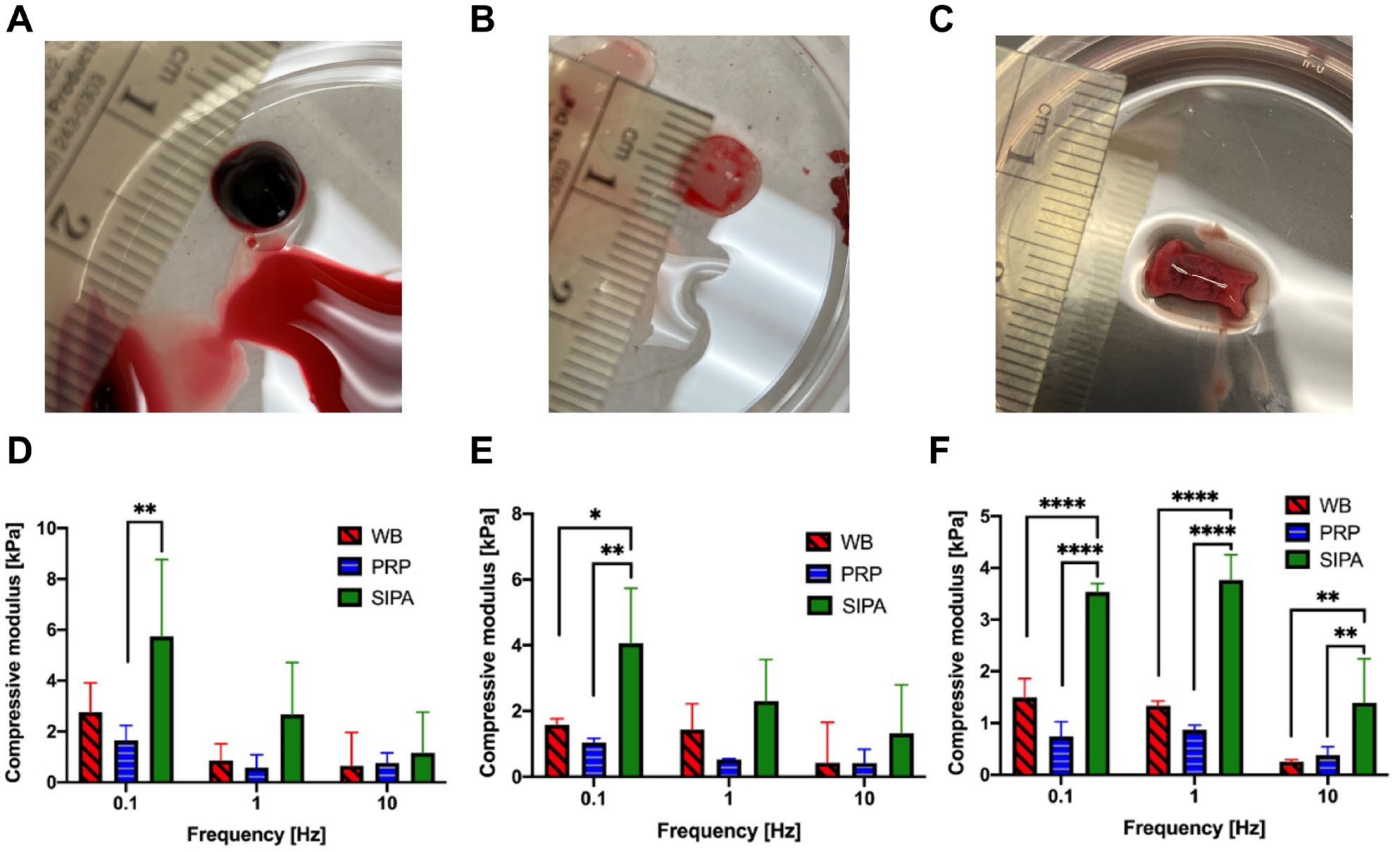
Dynamic Mechanical Analysis (DMA) on different clot types: (**A**) Whole blood (WB) coagulation clot, (**B**) Platelet Rich Plasma (PRP) coagulation clot, and (**C**) Shear-Induced Platelet Aggregation (SIPA) clot. (**D**–**F**) Blood clot compressive modulus measured by DMA. On average, all clots had an initial height of 1.8 mm. Three different displacements were applied on the blood clots. (**D**): 0.1 mm. (**E**): 0.2 mm. (**F**): 0.5 mm. The SIPA clots showed a two-fold higher modulus (2.9 ± 1.9 kPa, n = 3) compared to the WB (1.2 ± 0.9 kPa, n = 3) and PRP (0.8 ± 0.5 kPa, n = 3) coagulation clots as averaged for 3 different frequencies seen in Fig. 4F. *p < 0.05; **p < 0.01; ***p < 0.001; ****p < 0.0001.

To understand how clots can withstand cyclic blood pressure and resulting stresses, Dynamic Mechanical Analysis was conducted on (1) coagulation clots with RBCs (WB) and (2) without RBCs (PRP) as well as (3) SIPA clots (Fig. 4). Three different displacements (0.1 mm, 0.2 mm, and 0.5 mm) and three different frequencies (0.1 Hz, 1 Hz, and 10 Hz) were applied to each clot. All clots showed elastic behavior with a small phase difference in 0.1–1 Hz range (Fig. 4) but displayed a viscoelastic response to 10 Hz displacements. The SIPA clots demonstrated the highest compressive modulus for all conditions compared to the WB and PRP coagulation clots (Fig. 4). Note the statistical differences varied by strain shown in Fig. 4. However, on average, the SIPA clot (2.9 ± 1.9 kPa) had a 2.4 times higher compressive modulus than the WB coagulation clot (1.2 ± 0.9 kPa) and a 3.7-fold higher compressive modulus than the PRP coagulation clot (0.8 ± 0.5 kPa, p < 0.0001). And there was no statistical difference between coagulation clots with or without RBCs (WB vs. PRP).

### Clot deformation and critical lengths for the arterial occlusion

**Figure 5:**
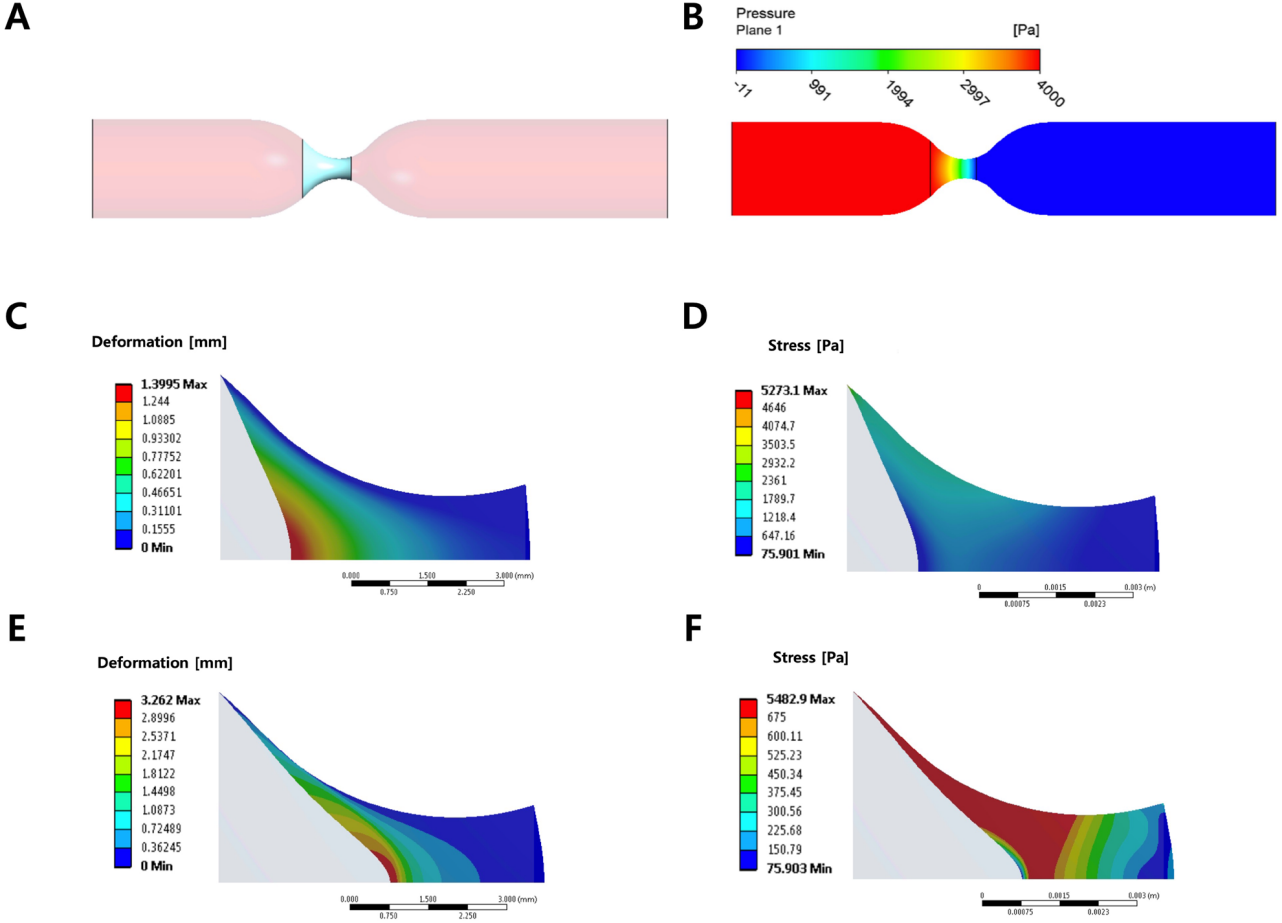
Computational analysis for flow conditions (CFD) and stress acting (FE) on clots. (**A**) A porous media CFD model with a clot (light blue) located at the stenosis. (**B**) Pressure contour by CFD in a stenosed tube with a blood clot. (**C**) Shear-Induced Platelet Aggregation (SIPA) clot FE result showed a maximum deformation of 1.4 mm. The undeformed state is shown as a gray contour with a flat entrance represented by the left edge of the figures. (**D**) SIPA clot FE result showed stress less than its breakage strength (4.6 kPa) throughout the clot. (**E**) WB coagulation clot FE result showed a maximum deformation of 3.3 mm. Undeformed state is shown as gray contour. (**F**) WB coagulation clot FE result showed more than 39% area having a stress higher than its breakage strength (675 Pa, shown in red).

An occlusive clot formed in a vessel needs to resist blood pressure to stop blood flow. While flow resistance increased tremendously with thrombus formation, flow did not cease entirely. Some microflow was observed through the microporous thrombus. This microflow from high pressure gradients can be interpreted as the permeability of the thrombus. A computational fluid dynamics analysis was performed to calculate the flow field around a clot after occlusion (Fig. 5A). The SIPA clot and WB coagulation clot were modeled as a porous media with different permeability (0.3 μm^2^ vs. 0.0005 μm^2^) and porosity (37% vs. 25%)^32^. The difference in permeability did not significantly change pressure distribution, less than 1 Pa due to pressure boundary conditions. However, SIPA clot and WB coagulation clot had significantly different flow rates through clots (5000 vs. 13 pL/min, respectively) and maximum blood velocity through the clot (2.2 m/s vs. 0.5 μm/s, respectively).

The calculated pressure distribution (Fig. 5B) was transferred for a finite element analysis to quantify clot deformation and stress distribution. The SIPA clot showed a maximum deformation of 1.4 mm at the center (Fig. 5C) and a peak tensile stress of 5 kPa at a singular point where the clot is attached to the wall (Fig. 5D). Apart from the tip, the SIPA clot areas had stress magnitudes less than the breakage strength (4.6 kPa, no red zone in Fig. 5D). The morphology after the deformation was similar to the histologic image described in the paper by Kim and Ku^30^. In contrast, the WB coagulation clot would theoretically deform more than 3 mm at the center, making the clot quite distorted (Fig. 5E). The WB coagulation clot subjected to arterial pressures would experience stress levels far beyond its breakage strength (675 Pa) as shown as a large red area in Fig. 5F; thus, it could not occlude the channel for long durations and would break off under arterial pressures.

### A small SIPA clot can occlude stenosed arteries

Mechanical strength directly relates to how a clot can occlude a stenosed artery. Silver, Baroldi, and Mariani^40^ found occlusive thrombosis most often occurred in 70–89% stenosed (by diameter) coronary artery. In addition, they found an occlusive thrombus can form within a 5 mm length stenosis. Assuming a rigid clot, 175 mmHg blood pressure^2^, and using breakage strengths obtained through this study, a critical length that would be required for a clot to occlude a stenosed coronary artery was calculated (Table 2). A SIPA clot would have sufficient strength to occlude a 60% stenosed (by diameter) coronary artery with a clot length of only 2 mm. In contrast, a WB coagulation clot would need a length longer than 14 mm to occlude the artery. The WB coagulation clot would then need to have a stagnant zone of 14 mm (3.5 × the diameter) to allow the fibrinogen to convert to fibrin *before* occlusion, a highly unlikely scenario.

**Table 2:** Critical clot length for occlusion based on the breakage strength of the thrombus in a nominal 4 mm coronary artery with a systolic pressure of 175 mmHg.

mm	Stenosed coronary by diameter [%]					
	40	50	60	70	80	90
Stenosis diameter	2.4	2.0	1.6	1.2	0.8	0.4
SIPA clot length	3.0	2.5	2.0	1.5	1.0	0.5
WB coagulation clot length	22.2	18.5	14.8	11.1	7.4	3.7

Clinical STEMI occlusions have been reported for 40% stenoses.

## Discussion

We generated in vitro blood clots and the mechanical testing results indicate that a SIPA clot has 7 times higher breakage strength (4.6 kPa vs. 0.6 kPa, p < 0.05) and 3 times higher stiffness (3.2 kPa vs. 1.2 kPa, p < 0.001) than a WB coagulation clot. The high mechanical strength of a SIPA clot is a key feature that allows it to stabilize in the high pressure and high shear rate environment for artery occlusion. The SIPA clot may have more strength than WB coagulation clots from the components or the architecture. We assume that protein strands of VWF and fibrin are likely similar in strength^41^. However, the number of bonds to many different connecting fibers may create stronger SIPA clots. Biophysics models of SIPA aggregation indicate that multiple strands of VWF with A1 roll up individual platelets with GPIa into dense agglomerates^42,43^. Estimates of the number of VWF-platelet bonds needed to create this strength is is easily accommodated by the 20,000 GPIb integrins on a platelet. Platelets have actin fibers that are likely much stronger than the floppy cell wall of RBCs. In contrast, WB coagulation clots require both conversion of fibrinogen to fibrin as well as completion of fibrin stabilization by Factor XIII, while platelets are sparce^44,45^. Thus, the seven-fold increase in strength likely stems from the architecture of greater numbers of VWF tethers bound by strong platelets forming the SIPA aggregate. Alternatively, the interface between components rather than the component itself may break off. Lam et al.^46^ did a single platelet measurement by using atomic force microscopy cantilever coated with fibrinogen, and found a rupture occurred at the interface when stress reached only 5 kPa. Meanwhile, Wellings and Ku^31^ suggested that a VWF net can capture a platelet under high shear via numerous Gp1b-A1 bonds that have strength of ~  < 100 pN. A concave VWF net can create 11,280 bonds, which can be converted to a strength of 100 kPa for the interface stress, a value 20 × that of fibrin. Moreover, more GPIIb/IIIa bonds can form and give additional strength between platelet-VWF interface after a platelet activation. Thus, the high strength in SIPA may derive from the increased total number of bonds in the aggregate. DMA analysis showed no statistical difference between WB and PRP coagulation clots, implying fibrin might contribute more to the coagulation clot mechanics compared to RBC.

With the measured stiffnesses of a SIPA clot, our FE model showed that the center of SIPA thrombus facing the flow deforms the same (1.4 mm) compared to histologic images (1.2 mm)^30^ and can block the large stenotic glass tube. In contrast, a WB coagulation clot would deform more than 3 mm. This large deformation would create very high stresses over a large area that exceeds its breakage strength. Using our calculated breakage strengths of blood clots, a critical length for a clot to occlude stenosed coronary artery was estimated and compared to clinical data^2,40^. Thus, a SIPA clot would be strong enough to resist an arterial blood pressure (175 mmHg) and occlude the artery with a length of just 2 mm. In contrast, blood would have to be stagnant over 14 mm to create a WB coagulation clot long enough before occluding a ~ 60% stenotic coronary. It is unlikely that this long a WB coagulation clot would form with the fast velocities before occlusion and is not typically seen imaging of ischemic stroke patients^47^. However, a WB coagulation clot may form after the SIPA clot occludes an artery, proximal and distal to the SIPA clot and make a clot longer than 14 mm^48^. Thus, a pathological post-mortem specimen may be mixture of SIPA clot and WB coagulation clot, especially if there was more than an hour time delay between the occlusion and a clot retrieval. A similar argument can be made for larger carotid arteries. This may explain why people have reported finding WB coagulation clots in coronary arteries^49^ or carotid arteries^50^ after late harvest making it hard to distinguish the real culprit of an occlusion.

There are reports of low grade stenoses leading to myocardial infarction^51^. These appear to respond better with agents targeting platelets^52^. Our analysis would support that occlusions in low grade stenoses must be due to the stronger platelet-rich thrombi (< 3 mm) as the WB coagulation clots would have to be very long (> 18 mm) to stop arterial blood flow (see Table 2 Critical clot lengths for 40% and 50% stenoses).

The mechanical strengths from this study are useful values for designing thrombectomy devices. Previous simulation models^53,54^ and in vitro assays^4,6,54,55^ used only WB coagulation clots to test thrombectomy devices. The mechanical strength of the SIPA clot quantified in the present study could be used to optimize wire-based thrombectomy devices to dislodge much stronger thrombi (e.g., stent and coil retriever). Although due to high variabilities observed in previous studies, validation of the SIPA clot stiffness from different blood types is desirable in future studies.

Our measurements show that a SIPA thrombus has a permeability of 0.3 μm^2^ and porosity of 37%. This porosity may contribute to the thrombus’ ability to withstand the high pressures without breaking. Both Welsh et al.^56^ and Du et al.^32^ have performed computational simulations of flow through thrombi and hypothesized that high permeability is important to clot stability. Our experimental measurements of thrombus permeability are in line with their predictions.

Limitations: Compared to Riha et al.^23^, the measured average value of breakage strength (0.3 kPa vs. 0.6 kPa) and stiffness (0.4 kPa vs. 1.2 kPa) of WB coagulation clots were higher in our study . Riha et al. tested WB coagulation clots immediately after mixing calcium chloride, thus fibrin polymerization could have been immature to provide a higher strength; whereas, our study allowed 30 min to 1 h for the WB or PRP coagulation clots to be polymerized and platelets to contract^46^. For clot generations, we used citrate for WB or PRP coagulation clots and heparin for SIPA clots. Different anticoagulants may affect mechanical properties of clots. For the DMA analysis, although we gained partial statistically significant differences, we may find more differences with sample size larger than three. Lastly, we simulated deformation of a WB coagulation clot that show stresses that are higher than strength, but this needs experimental validation.

## Conclusion

Compared to WB coagulation clots, in vitro SIPA clots are two-fold stiffer and seven-fold stronger. In vivo clots may not have the same strengths. The measured strength allows thrombi to occlude an artery at high systolic systemic pressures. Such strong SIPA thrombi can also form under the high shear conditions of a stenotic artery. In contrast, a WB coagulation clot is a material that is too weak to create an arterial occlusion and would require long stagnation times for formation. Due to its distinctive mechanical properties, the SIPA clot may require optimized thrombectomy devices for therapies to establish reperfusion or a new thrombolytic agent aimed at platelet-VWF aggregates.

### Supplementary Information

Supplementary Figures.
