# Supplementary material for: Material strengths of shear-induced platelet aggregation clots and coagulation clots
Source: Sci Rep. 2024 May 20;14:11460. doi: 10.1038/s41598-024-62165-1 (PMC11106319; doi:10.1038/s41598-024-62165-1)
Supplement: Supplementary file 1 — Supplementary Figures. [file 41598_2024_62165_MOESM1_ESM.docx]

**Supplementary Materials**


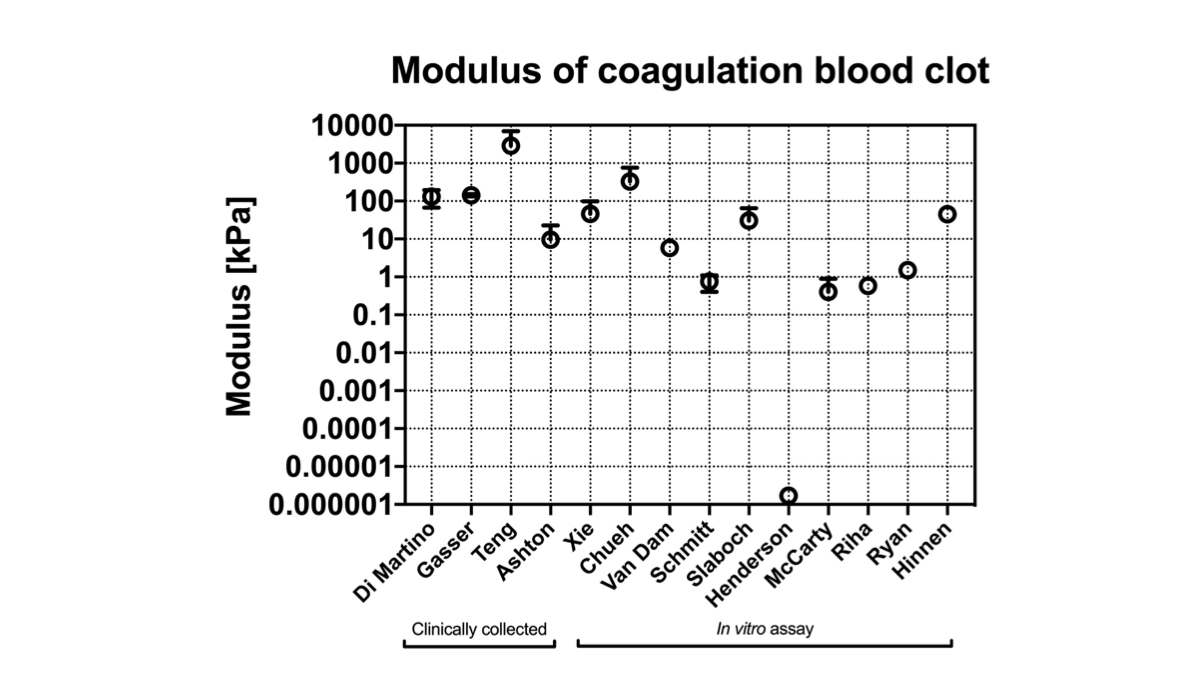


**Suppl Fig. 1** Coagulation blood clot modulus from Johnson et al. (2017) and Slaboch et al. (2012).

**
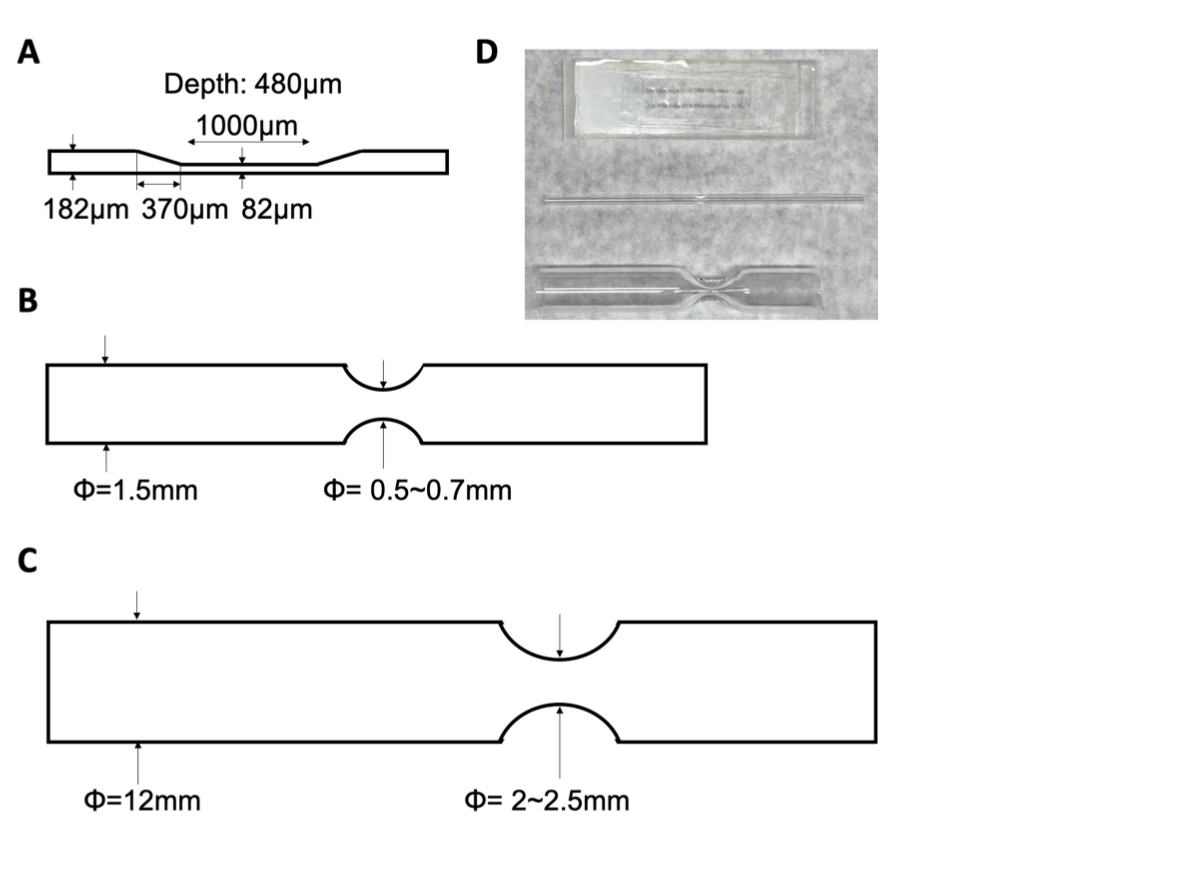
**

**Suppl Fig. 2** Geometry of the stenotic chambers: (A) Microfluidic chamber (55% stenosis by diameter), (B) Capillary glass tube (ID = 1.5 mm, 65% stenosis by diameter), and (C) Large glass tube (ID = 12 mm, 80% stenosis by diameter). (D) Photograph of actual test sections showing the size comparison of the three chambers.

**
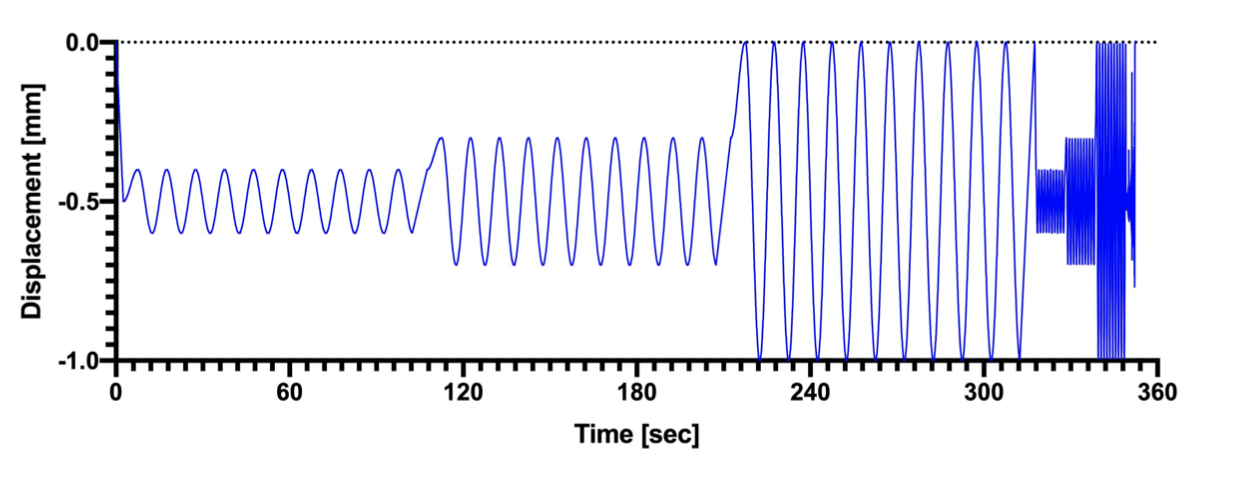
**

**Suppl Fig. 3** Input displacements and frequencies for DMA of the blood clots.


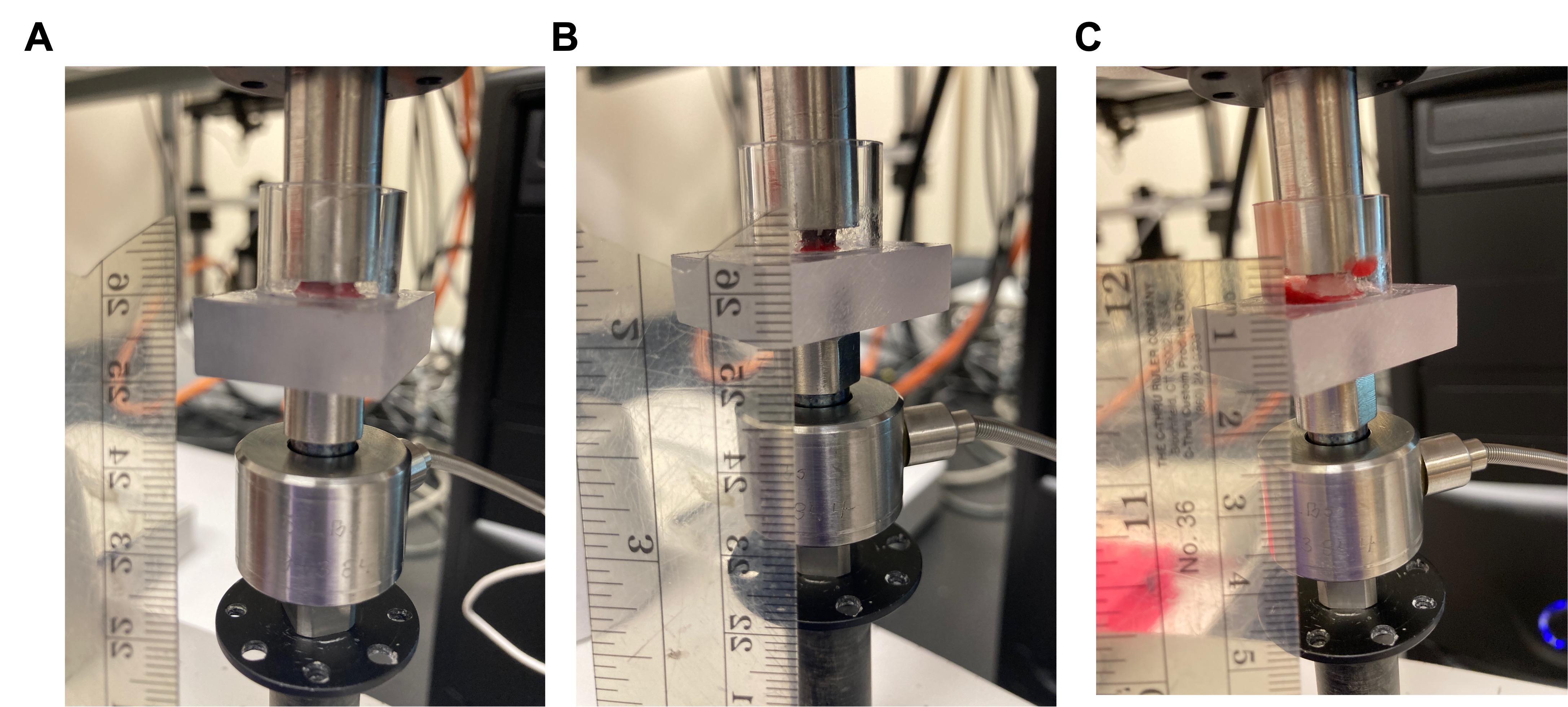


**Suppl Fig. 4** Blood clots placed in a chamber for DMA (A: SIPA clot, B: Whole blood coagulation clot, C: PRP coagulation clot).

**
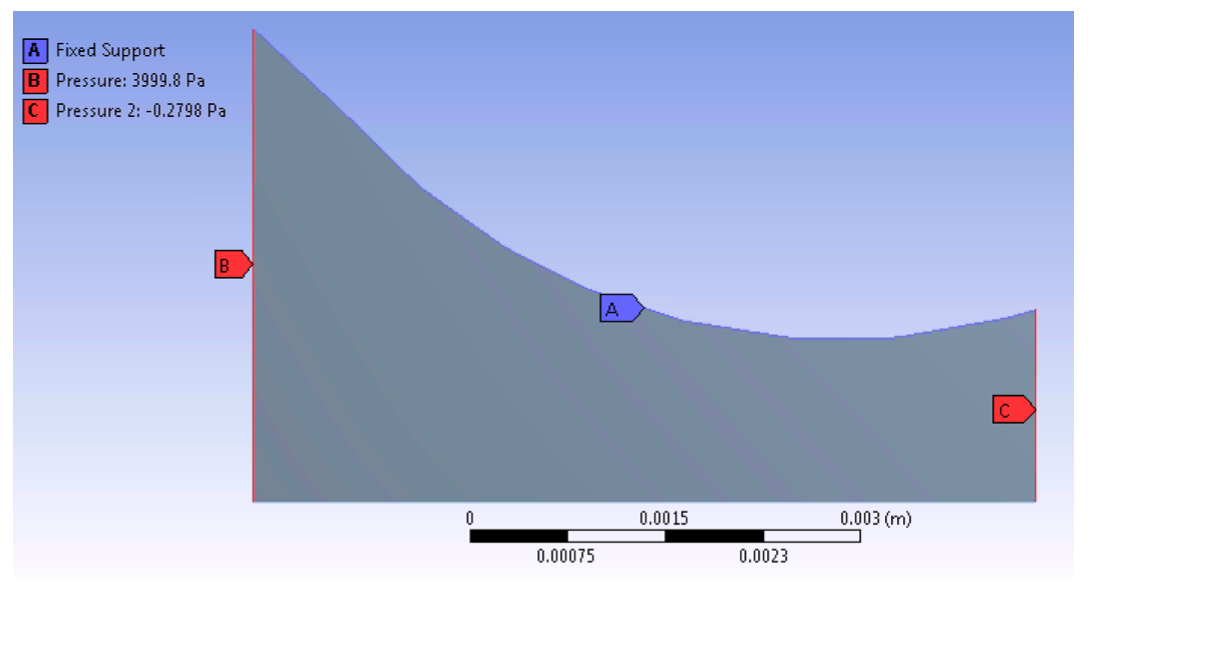
**

**Suppl Fig. 5** A 2-D axisymmetric model for a simulation of clot deformation and stress distribution. (A) A fixed support boundary condition was applied at the interface between the clot and channel wall. Calculated pressure was applied on (B) proximal and (C) distal clot surfaces.
